# Validating a novel natural language processing pathway for automated quality assurance in surgical oncology: incomplete excision rates of 34 955 basal cell carcinomas

**DOI:** 10.1093/bjs/znad055

**Published:** 2023-03-20

**Authors:** Stephen R Ali, Thomas D Dobbs, Matthew Jovic, Huw Strafford, Beata Fonferko-Shadrach, Arron S Lacey, Namor Williams, William Owen Pickrell, Hayley A Hutchings, Iain S Whitaker

**Affiliations:** Reconstructive Surgery and Regenerative Medicine Research Centre. Institute of Life Sciences, Swansea University Medical School, Swansea, UK; Welsh Centre for Burns and Plastic Surgery, Morriston Hospital, Swansea, UK; Reconstructive Surgery and Regenerative Medicine Research Centre. Institute of Life Sciences, Swansea University Medical School, Swansea, UK; Welsh Centre for Burns and Plastic Surgery, Morriston Hospital, Swansea, UK; Reconstructive Surgery and Regenerative Medicine Research Centre. Institute of Life Sciences, Swansea University Medical School, Swansea, UK; Neurology and Molecular Neuroscience Group, Institute of Life Science, Swansea University Medical School, Swansea University, Swansea, UK; Health Data Research UK, Swansea University Medical School, Swansea University, Swansea, UK; Neurology and Molecular Neuroscience Group, Institute of Life Science, Swansea University Medical School, Swansea University, Swansea, UK; Health Data Research UK, Swansea University Medical School, Swansea University, Swansea, UK; Neurology and Molecular Neuroscience Group, Institute of Life Science, Swansea University Medical School, Swansea University, Swansea, UK; Health Data Research UK, Swansea University Medical School, Swansea University, Swansea, UK; Department of Cellular Pathology, Morriston Hospital, Swansea, UK; Neurology and Molecular Neuroscience Group, Institute of Life Science, Swansea University Medical School, Swansea University, Swansea, UK; Department of Neurology, Morriston Hospital, Swansea, UK; Patient and Population Health and Informatics Research, Swansea University Medical School, Swansea, UK; Reconstructive Surgery and Regenerative Medicine Research Centre. Institute of Life Sciences, Swansea University Medical School, Swansea, UK; Welsh Centre for Burns and Plastic Surgery, Morriston Hospital, Swansea, UK

## Introduction

Accurate and accessible outcomes following a cancer diagnosis are crucial in maintaining robust quality assurance. Multidisciplinary team (MDT) meetings aim to improve care through group consensus, national guidance, clear documentation, and communication. However, research has highlighted limitations in their outputs, especially regarding the way outcomes are databased and audited^1^. Novel technologies, such as artificial intelligence (AI), have the potential to improve this, as cited in the Royal College of Surgeons of England ‘Future of Surgery’ commission^2^.

Natural language processing (NLP), a form of AI, offers a novel approach to automate extraction of detailed clinical information from unstructured electronic healthcare record data, such as clinic letters, operative notes, and histopathology reports. In a recent systematic review, NLP was found to have higher sensitivity and comparable specificity in identifying postoperative complications compared to conventional administrative methods^3^.

To date, no studies have used NLP to determine incomplete excision rates in surgical oncology. In this study, the feasibility of automatically extracting and interpreting margin status from histopathology reports using an NLP-based system was demonstrated.

## Methods

A multicentre, pan-specialty, retrospective analysis of consecutive patients with histologically confirmed basal cell carcinoma (BCC) managed with surgical excision and examined using the bread loafing cross-section technique was undertaken. The study period covered a 17-year period from 2004 to 2021. Cases were identified from InterSystems TrakCare Laboratory Information Management System (InterSystems TrakCare Lab, Cambridge, Massachusetts, USA), using SNOMED RT codes for BCC. Primary, recurrent and previously excised lesions were grouped together for analysis. Diagnostic biopsies were excluded.

Free-text pathology reports were retrieved and saved in text file format. These were then processed using a previously validated and published rule-based NLP information extraction system^4^. Comma-separated variable (CSV) text files were generated from the respective canonical subheadings of the pathology report.

**Fig. 1:**
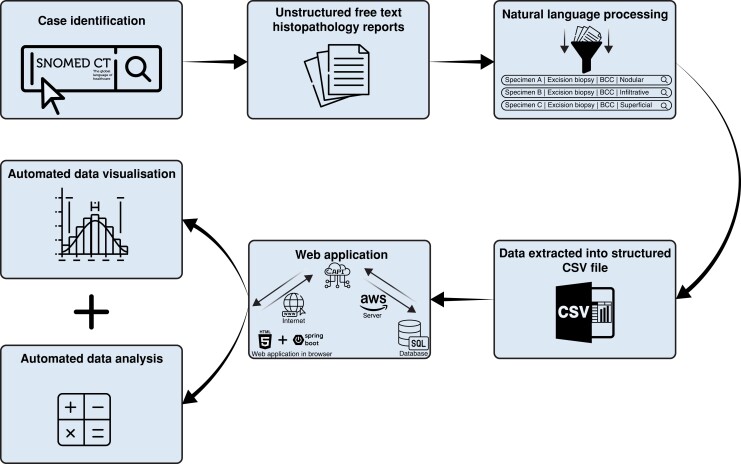
Schematic representation of our automated population-based quality assurance model BCC, basal cell carcinoma.

A Java™ Spring Boot (VMware, Palo Alto, California, USA) web application hosted on Amazon Web Services (Amazon.com, Seattle, Washington, USA) in EC2 was then developed. Respective CSV files were imported into a relational database management system. The process for generating incomplete excision rates is shown in *Fig. 1*.

A range of tumour, patient, and surgical factors were recorded. The British Association of Dermatologists’ (BAD) adaption of the National Comprehensive Cancer Network guidelines on the treatment of BCC (*Table S1*) was modified to categorize BCCs clinicopathologically into low and high risk (*Fig. S1*)^5^. The primary endpoints were histological margin status, risk status, and speciality of the operating surgeon. The margin status was defined as either clear (≥1 mm) or involved (0 mm).

Sample size for the internal validation cohort was determined by conducting an a priori calculation. Retrospective analysis of the baseline variables and outcomes was undertaken retrospectively by two independent and blinded expert clinicians. This clinical pathway served as the reference standard for the study. Disagreements were resolved by case discussion until a consensus was reached. The single-consensus clinician-derived outputs were then compared against NLP-derived outputs for analysis. Percentage agreement and Cohen’s kappa were used as measures of agreement between NLP-derived and clinician-derived completeness of excision, risk status, and speciality of the operating surgeon. Statistical analysis was done in R version 4.1.1 (R Core Team; R Foundation for Statistical Computing, Vienna, Austria).

Ethical committee approval was obtained from Swansea University Medical School Research Ethics Subcommittee (reference no: 2020-0025). The study was performed in accordance with the Declaration of Helsinki. Data are reported following Transparent Reporting of a multivariable prediction model for Individual Prognosis or Diagnosis (TRIPOD).

## Results

Some 34 955 lesions in 15 657 patients were included. Baseline characteristics are shown in *Table S2*. The overall incomplete excision rate in this cohort was 5.5 per cent.

The incomplete excision rate stratified by risk and margin by the web application is shown in *Table 1*. There were 6152 histopathology reports in the validation cohort, assessing accuracy of completeness of excision, risk status, and speciality of operating surgeon. There was 0.99 agreement (95 per cent c.i. 0.98 to 0.99; Cohen’s kappa = 0.74 (95 per cent c.i. 0.68 to 0.80); *P* < 0.001) for completeness of excision. There was 0.99 agreement (95 per cent c.i. 0.99 to 0.99; Cohen’s kappa = 0.73 (95 per cent c.i. 0.69 to 0.77), *P* < 0.001) for speciality of the operating surgeon.

**Table 1:** Incomplete excision rates for all specialities stratified by risk (high or low) and margin (peripheral or deep)

Risk status	Peripheral margin incomplete rate (%)	Deep margin incomplete rate (%)	Overall incomplete rate (%)
Low risk	0.9	1.2	2.1
High risk	1.7	1.7	3.4
Total	2.6	3.0	5.5

Using a MacBook M1 Pro with 16 GB RAM, the NLP pipeline extracted and structured 2 184 309 items of information in 22.7 min, a rate of 689.7 cases/min. A single clinician could extract data at a rate of 0.25 cases/min on the validation cohort. Extrapolating this rate to 15 657 histopathology reports, it would take a clinician 29.8 weeks (8 h/day, Monday to Friday, with a 30-min rest break every 4 h) to extract the same amount of data, representing a time saving of 208 days.

Post-hoc binary logistic regression showed that plastic surgeons were more likely to achieve clear margins than other specialties (*Table 2*). The incomplete excision rates were calculated by converting log odds from the model into probabilities on a 0 to 1 scale for significant values. Probabilities were then converted into incomplete excision rates (*Table 2*). This approach accounts for risk when comparing specialties, rather than using raw incomplete excision rates.

**Table 2:** *Post-hoc* binary logistic regression and probabilistic modelling

Specialty	Post-hoc binary logistic regression to model the relationship between uninvolved margin and speciality			Incomplete excision rate across specialities stratified by risk status	
	Odds ratio (95% c.i.)	Incomplete excision rate (%) (95% c.i.)	*P value*	High risk incomplete excision rate (%)	Low risk incomplete excision rate (%)
Oral and maxillofacial surgery	0.62 (0.52–0.75)	7.78 (6.96–8.60)	<0.001	8.23	6.28
Other	0.57 (0.46–0.70)	8.28 (7.30–9.27)	<0.001	8.96	6.86
Dermatology	0.53 (0.47–0.61)	8.26 (7.16–9.37)	<0.001	9.50	7.28
Ear, nose, and throat surgery	0.43 (0.33–0.56)	11.09 (10.08–12.11)	<0.001	11.56	8.90
General practice	0.22 (0.17–0.29)	17.47 (15.41–19.53)	<0.001	20.22	15.93
Ophthalmology	0.21 (0.17–0.27)	18.29 (16.12–20.46)	<0.001	20.79	16.40
General surgery	0.00	0.00	0.934	-	-

Plastic surgery as reference specialty. Risk as covariate. Note the incomplete excision rate in the general surgery cohort was 0 per cent. Conversion of each log odds was undertaken on significant values only.

## Discussion

In this study, an automated population-based approach to quality assurance in surgical oncology was validated, using NLP to extract margin status from histopathology reports at scale in the most common human cancer. A web application was used to automate the analysis of incomplete excision rates, stratifying margin and risk before undertaking post-hoc analysis to investigate the relationship between uninvolved margin and speciality. As shown, it is possible to reach high levels of percentage agreement (> 90 per cent) when comparing the NLP-based method to blinded expert clinicians. This is also in a tumour type that, traditionally, has poor compliance with minimum data set reporting, suggesting that for other tumours, agreement would likely be similar or higher. The rate of incomplete excision (5.5 per cent) in this study is comparable to joint National Institute for Health and Care Excellence (NICE) and BAD guidance, with a target rate of ≥95 per cent for complete excision^6^. This suggests that the output of this algorithm is valid.

Although this innovative approach allows for automated, rapid, and large-scale analysis of health data, saving significant time and resources, there remains an argument for integrating a ‘human in the loop’ at the MDT level to ensure the highest level of accuracy and patient care. The present work aligns with the vision outlined by the Topol Review, which foresees AI and other digital technologies augmenting the capabilities of healthcare professionals rather than replacing them, allowing them to focus on providing the best possible care to patients^7^.

This work is novel, with a recent systematic review for NLP highlighting that only one other model was able to extract tumour margin status in a small test set. There are no reports of using NLP at a population level for quality assurance in surgical oncology. Furthermore, this study represents a large global series of incomplete BCC excision rates. This was achieved in an infinitesimally smaller time frame than would be possible with human extraction and processing.

The system developed here allows for the rapid and accurate assessment of a number of parameters to which clinicians, MDTs, and service providers can be assessed. This tool could be used for standard benchmarking, confidential feedback to service providers and users, and for freeing up healthcare professional time in MDTs and administrative tasks to focus on delivering high-quality patient care.

## Supplementary Material

znad055_Supplementary_Data

## Data Availability

All data were anonymised prior to collection. We do not have a data-sharing agreement for the data; however, we are exploring ways of obtaining patient consent and endeavour to produce a minimum data set for cross-platform testing.
