# Supplementary material for: Validating a novel natural language processing pathway for automated quality assurance in surgical oncology: incomplete excision rates of 34 955 basal cell carcinomas
Source: Br J Surg. 2023 Mar 20;110(9):1072–5. doi: 10.1093/bjs/znad055 (PMC10416688; doi:10.1093/bjs/znad055)

**Validating a novel natural language processing pathway for automated quality assurance in surgical oncology: incomplete excision rates of 34,955 basal cell carcinomas**

Stephen R Ali ^1, 2^, Thomas D Dobbs ^1, 2^, Matthew Jovic ^1^, Huw Strafford ^3, 4^, Beata Fonferko-Shadrach ^3,4^, Arron S Lacey ^3, 4^, Namor Williams ^5^, William Owen Pickrell ^3, 6^, Hayley A Hutchings ^7^, Iain S Whitaker ^1, 2^

1. Reconstructive Surgery and Regenerative Medicine Research Centre. Institute of Life Sciences, Swansea University Medical School, Swansea, UK
2. Welsh Centre for Burns and Plastic Surgery, Morriston Hospital, Swansea, UK
3. Neurology and Molecular Neuroscience Group, Institute of Life Science, Swansea University Medical School, Swansea University, Swansea, UK
4. Health Data Research UK, Data Science Building, Swansea University Medical School, Swansea University, Swansea, UK
5. Department of Cellular Pathology, Morriston Hospital, Swansea, UK
6. Department of Neurology, Morriston Hospital, Swansea, UK
7. Patient and Population Health and Informatics Research, Swansea University Medical School, Swansea, UK

**Corresponding author:**

Mr Stephen Ali BM MMedSc(Hons) PGCME MAcadMEd FHEA MRCS(Eng)

Reconstructive Surgery & Regenerative Medicine Research Centre, Institute of Life Sciences, Swansea University Medical School, Swansea SA2 8PP, United Kingdom

Tel: 01792205678

Email: stephenrahemali@gmail.com

ORCID: https://orcid.org/0000-0002-9917-3432

**Supplementary Materials - Index**

| **Supplementary Figures and Tables** |  |
| --- | --- |
| Table S1 | *pag. 2* |
| Table S2 | *pag. 3* |
| Figure S1 | *pag. 4* |
|  |  |

**Supplementary Figures and Tables**

**Table S1:** Criteria for low-risk and high-risk BCC.

| **Tumour, patient and surgical variable collected** | **Low risk** | **High risk** |
| --- | --- | --- |
| Location and size | Area A ≤ 20 mm (maximum clinical diameter) | Area A > 20 mm (maximum clinical diameter) |
|  | Area B ≤ 10 mm (maximum clinical diameter) | Area B > 10 mm (maximum clinical diameter) |
|  |  | Area C |
| Borders | Well defined | Poorly defined |
| Primary vs. recurrent | Primary | Recurrent |
| Immunosuppression | No | Yes |
| Site of prior radiotherapy | No | Yes |
| Growth pattern | Nodular, cystic, superficial, fibroepithelial | Infiltrative (infiltrating, morphoeic, micronodular and multinodular) |
| Differentiation: basosquamous | Absent | Present (with or without lymphovascular invasion) |
| Level of invasion | Dermis, subcutaneous fat | Beyond subcutaneous fat |
| Depth (thickness) | ≤ 6 mm | > 6 mm |
| Perineural invasion | Absent | Present |
| Pathological TNM stage | pT1 | pT2 |

TNM, Tumour–Nodes–Metastasis. One or more criteria satisfies the criteria for high-risk.

**Table S2:** Baseline characteristic of included patients.

| **Specialty** | **High risk**  **(n = 8855)** | **Low risk**  **(n = 6802)** | **Total**  **patients** | ***p value*** |
| --- | --- | --- | --- | --- |
| Dermatology | 3581 | 4546 | 8127 | < 0.001 |
| Plastic surgery | 2648 | 951 | 3599 | < 0.001 |
| Oral and maxillofacial surgery | 1251 | 368 | 1619 | < 0.001 |
| Other | 589 | 308 | 897 | < 0.001 |
| Ear, nose and throat | 402 | 82 | 484 | < 0.001 |
| General practice | 174 | 292 | 466 | < 0.001 |
| Ophthalmology | 190 | 237 | 427 | < 0.001 |
| General surgery | 20 | 18 | 38 | 0.7456 |

*p*-values are for chi-square tests for differences in the proportion of high/low risk patients between speciality.

**Figure S1:** Topographical areas used for classification of low-risk and high-risk BCC that corresponds with Table 1.


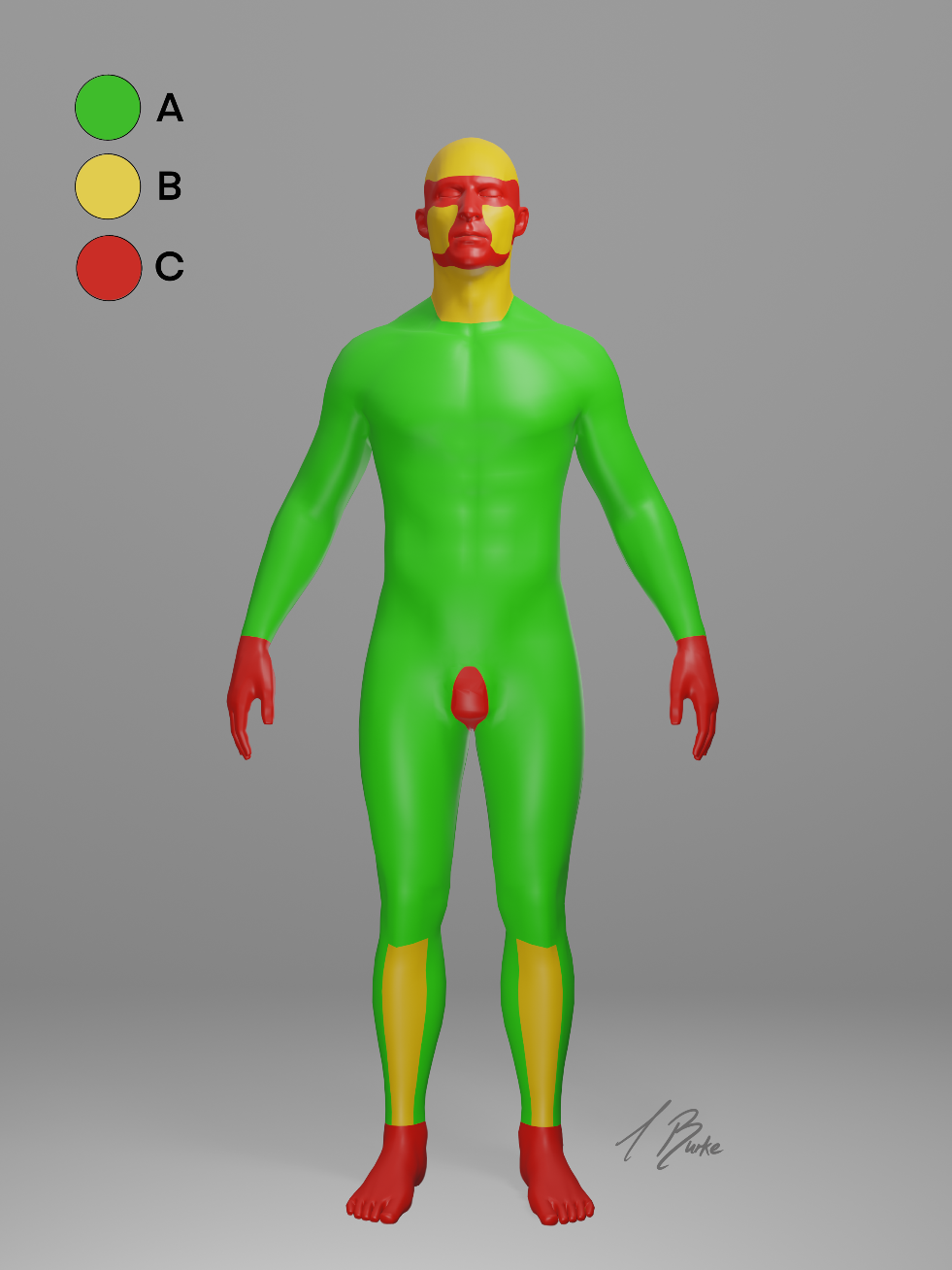

Supplement: znad055_Supplementary_Data [file znad055_supplementary_data.docx]
